# Functionalizing a Tapered Microcavity as a Gas Cell for On-Chip Mid-Infrared Absorption Spectroscopy

**DOI:** 10.3390/s17092041

**Published:** 2017-09-06

**Authors:** N. Pelin Ayerden, Julien Mandon, Frans J. M. Harren, Reinoud F. Wolffenbuttel

**Affiliations:** 1 Electronic Instrumentation Laboratory, Microelectronics Department, Faculty of EEMCS, Delft University of Technology, Mekelweg 4, 2628 CD Delft, The Netherlands; r.f.wolffenbuttel@tudelft.nl; 2 Life Science Trace Gas Research Group, Department of Molecular and Laser Physics, Institute for Molecules and Materials, Radboud University Nijmegen, Heijendaalseweg 135, 6525 AJ Nijmegen, The Netherlands; j.mandon@science.ru.nl (J.M.); f.harren@science.ru.nl (F.J.M.H.)

**Keywords:** gas sensors, optical absorption spectroscopy, mid-infrared, microcavities, Fabry–Perot, Fizeau interferometer

## Abstract

Increasing demand for field instruments designed to measure gas composition has strongly promoted the development of robust, miniaturized and low-cost handheld absorption spectrometers in the mid-infrared. Efforts thus far have focused on miniaturizing individual components. However, the optical absorption path that the light beam travels through the sample defines the length of the gas cell and has so far limited miniaturization. Here, we present a functionally integrated linear variable optical filter and gas cell, where the sample to be measured is fed through the resonator cavity of the filter. By using multiple reflections from the mirrors on each side of the cavity, the optical absorption path is elongated from the physical μm-level to the effective mm-level. The device is batch-fabricated at the wafer level in a CMOS-compatible approach. The optical performance is analyzed using the Fizeau interferometer model and demonstrated with actual gas measurements.

## 1. Introduction

Optical absorption spectroscopy is a widely used method for gas composition measurement due to self-referencing capability and nondestructive property [1]. Sensitivity to sample concentration and selectivity for different sample compositions are key performance parameters of this method. A long absorption path must be employed for measuring at a low concentration, while high spectral resolution and wide operating wavelength range are required for high selectivity.

The dimensions of a gas absorption spectrometer are predominantly determined by those of the gas cell. Multipass gas cells [2,3] and cavity-enhanced methods [4,5] are used in commercially available benchtop spectrometers to elongate the absorption path for trace gas detection, while selectivity is limited by the narrow bandwidth of the light source.

On-chip implementations of optical absorption spectrometers aim to achieve selectivity and sensitivity comparable to their benchtop counterparts, in a small footprint [6]. Despite their wide wavelength range, Fourier transform infrared (FTIR) spectrometers based on miniaturized interferometers still require an external gas cell to enhance sensitivity [7,8]. Microring [9] and microdoughnut [10] resonators elongate the absorption path beyond the physical dimensions of the structure; however, their spectral response is susceptible to fabrication tolerances. Disordered photonic chips can achieve sub-nm resolution by exploring scattering patterns, but suffer from a limited wavelength range [11]. Despite the improved resolution, wavelength-selective devices using digital planar holography [12,13], photonic crystal slot waveguide [14], microring resonator combined with a diffraction grating [15,16], multimode spiral waveguide [17], cylindrical Fabry–Perot (FP) cavity [18,19] and double ring resonator [20] are suitable to be built laterally on a wafer, which complicates on-chip integration with the light source and the detector.

Combining a detector array with wavelength-selective channels formed by either fixed planar FP filters [21] or colloidal quantum dots [22] allows for bottom-up wafer-level integration. However, the spectral performance is highly sensitive to the resonator thickness in the former and the nanocrystal size in the latter. A linear variable optical filter (LVOF) that is composed of one flat and one slightly tilted mirror with a tapered resonator in-between is less affected by fabrication errors due to its continuous structure. Combined with a detector array, a high resolution wideband spectral response can be measured with an LVOF.

Here, we present the gas-filled LVOF, where the resonator cavity of the filter is also used as a gas cell. The high-order operation and highly reflective mirrors ensure a mm-level effective optical path from a microcavity; thus, high sensitivity, while the high resolution and the tapered cavity provide selectivity in a wide wavelength range. Due to its CMOS-compatible bottom-up fabrication approach, the gas-filled LVOF is highly suitable for monolithic integration with a detector array and electronics. Combined with a wideband source, it would be a paradigm-changing approach for on-chip spectroscopy.

## 2. Materials and Methods

**Figure 1:**
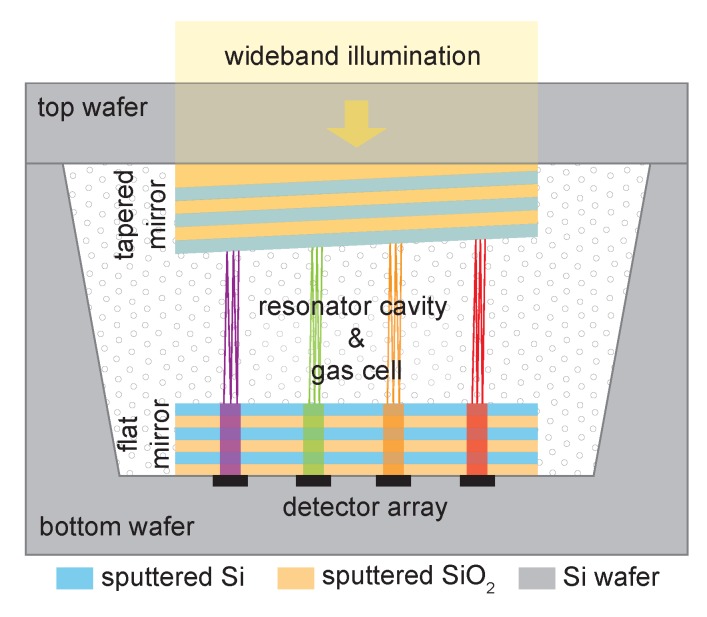
Side view of the gas-filled linear variable optical filter (LVOF), composed of a flat and a tapered mirror with a tapered resonator cavity in-between.

Operation of the gas-filled LVOF is based on the cavity-enhanced absorption spectroscopy (CEAS) method, which exploits high-finesse optical cavities to enhance the interaction between light and matter [23]. In contrast to the almost ideal mirror reflectivity in this method ($1-R∼10^{-4}$), the gas-filled LVOF employs mirrors with high reflectivity (R>0.95). The device is composed of one flat and one tapered Bragg mirror with a tapered resonator cavity in-between as shown in Figure 1. The resonator cavity also serves as a gas cell, where wavelength selection and optical absorption occur simultaneously. The wavelength to be transmitted through the filter is determined by the cavity length, i.e., the distance between the flat and the tapered mirror. Therefore, if the filter is fabricated on top of a detector array, each pixel measures a different wavelength. Furthermore, multiple reflections between the mirrors intensify the interaction between the light and the sample, thus elongating the effective optical absorption path beyond the physical dimensions of the cavity. When the cavity is filled with the sample, the intensity of the light transmitted through the filter decreases according to the spectral distribution of the sample absorption. 

The spectral resolution and the operating wavelength range of the gas-filled LVOF can be adjusted for different applications by altering the mirror materials and optical layer thicknesses. The wavelength range must be wide enough to cover the distinctive absorption features of the sample to be analyzed, while the spectral resolution must be high enough to distinguish the constituents of the sample. The device presented here is intended to distinguish hydrocarbons (i.e., the main components of natural gas), specifically methane (CH_4_), ethane (C_2_H_6_) and propane (C_3_H_8_) in the 3.2 μm to 3.4 μm wavelength range in the mid-infrared.

An enhanced effective optical path length is achieved by combining highly reflective mirrors and a resonator cavity with the maximum length that the selected operating wavelength range allows. The longest possible cavity varies from 24 μm to 25.5 μm for operation in the 3.2 μm to 3.4 μm wavelength range, corresponding to the 15th operating order. The Bragg mirrors are composed of three pairs of Si and SiO_2_ thin-film layers with quarter-wavelength optical thickness, while the first layer of the tapered mirror is varied linearly along the length of the filter to create the tapered cavity. The combined reflectivity of the mirrors varies between 98.4% and 99.5%, depending on the position along the length of the filter.

Bragg mirrors are composed of alternate layers of high and low refractive index materials with quarter-wavelength optical thickness, where the physical thickness is adjusted for the refractive index of the material. The constructive interference of the reflected beams, i.e., high mirror reflectivity, occurs when the quarter-wavelength optical thickness requirement is met. The position-dependency of the reflectivity of Bragg mirrors is caused by two factors. Firstly, the optical thickness requirement is realized for only a single wavelength for fixed layer thickness. The center design wavelength of 3.3 μm forces the mirror reflectivity to diverge from the optimum value at other wavelengths. Since different wavelengths are observed at different positions along the length of the device, the mirror reflectivity becomes position dependent. Secondly, the tapered SiO_2_ layer of the tapered mirror implies a varying thickness along the length of the filter and renders the mirror reflectivity position dependent. Therefore, the combined effect of the wavelength and the layer thickness dependency causes the mirror reflectivity to vary along the length of the device. It must be noted that variations caused by wavelength dependency are negligible compared to the variations caused by the tapered SiO_2_ layer.

LVOFs are conventionally treated as an array of FP filters composed of parallel mirrors with varying resonator thickness along the length of the device. However, in contrast to the constant value in the FP filter, the angle of reflection changes during propagation in an LVOF, due to the taper of the resonator. This causes the light beam to shift laterally and eventually walk off the wedge-shaped cavity after multiple reflections. With increasing cavity length and mirror reflectivity, the effect of walk-off on the spectral response of an LVOF can no longer be neglected. Instead of the FP approach, the LVOF must be regarded as a Fizeau wedge, in which the tilt angle between the mirrors on both sides of the resonator is taken into account [24].

**Figure 2:**
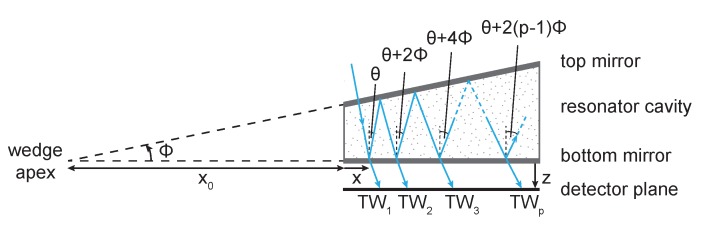
Propagation of a light beam in an LVOF.

A monochromatic plane wave at a wavelength of λ, entering the LVOF at an incidence angle of θ with respect to the flat bottom mirror undergoes multiple reflections as shown in Figure 2. The angle of reflection changes at every interaction with the top mirror based on the angle between the top and the bottom mirrors, ϕ, causing a position (x,z) and tilt angle (ϕ) dependent phase for each transmitted wave as given in Equation (1), where $\Delta S_{p}$ represents the the optical path difference between the first and the *p*th wave. The transmitted waves with different phases ($TW_{1},TW_{2},TW_{3},\ldots ,TW_{p}$) interfere constructively at the detector plane (*z*) at a position along the length of the filter (*x*) that fulfills the constructive interference condition for the wavelength of the input wave.

(1) $$\delta_{p}=\frac{2\pi }{\lambda }\underset{\Delta S_{p}}{\underset{︸}{\left(\begin{array}{c} x\left[\sin\left(\theta +2\left(p-1\right)\phi \right)-\sin\theta \right]\\ +z\left[\cos\left(\theta +2\left(p-1\right)\phi \right)-\cos\theta \right] \end{array}\right)}}$$

The intensity ratio of the transmitted to the input light, i.e., transmittance ($T_{LVOF}$), is calculated by the summation of infinitely many plane waves with different phases ($\delta_{p}$), whose amplitudes are adjusted by the combined reflectivity ($R_{m}$) and transmissivity ($T_{m}$) of the mirrors as given in Equation (2). The transmittance is calculated at every *x*-position throughout the entire length of the filter to achieve the spectral response of the LVOF to a single wavelength. The wideband transmission response of the filter is obtained by repeating this calculation at various wavelengths.

(2) $$T_{LVOF}=T_{m}^{2}{\left|\sum_{p=1}^{\infty }R_{m}^{\left(p-1\right)}e^{i\delta_{p}}\right|}^{2}$$

Attenuation of a wave due to optical absorption is determined by multiplying its initial amplitude by $e^{-\alpha cL}$, where α and *c* are the absorption coefficient and the concentration of the sample, and *L* is the optical absorption path length. The absorption coefficient is a unique wavelength-dependent property of a sample; however, a spectrometer with high resolving power could detect finer spectral features. The optical absorption path length, on the other hand, is independent of the sample and is defined as the physical length of the path that the light beam follows through the sample.

Adjusting the absorption coefficient according to the resolving power of the spectrometer and estimating the attenuation due to optical absorption are intertwined in a gas-filled LVOF, since wavelength selection and optical absorption occur simultaneously. Therefore, the following simulation sequence is employed. Firstly, the spectral distribution of a single wavelength is calculated along the length of the filter. Then, the *x*-position of the peak of the transmission curve is used for calculating the spectral response in the wavelength domain. This curve is convolved with the high-resolution absorption coefficient spectrum of the sample gas to include the effect of the unique shape and the resolution of the transmission curve of the LVOF in adjusting the absorption coefficient. Subsequently, the adjusted absorption coefficient is employed in calculating the response of the gas-filled LVOF using Equation (3), where the optical path difference between the transmitted waves is used as the optical absorption path length:

(3) $$\begin{array}{cc} T_{Gas-filled\;LVOF} & =T_{m}^{2}{\left|\sum_{p=1}^{\infty }R_{m}^{\left(p-1\right)}e^{i\delta_{p}-\alpha c\Delta S_{p}}\right|}^{2}\\ & =T_{m}^{2}{\left|\sum_{p=1}^{\infty }R_{m}^{\left(p-1\right)}e^{\left(i-\alpha c\frac{\lambda }{2\pi }\right)\delta_{p}}\right|}^{2}. \end{array}$$

**Figure 3:**
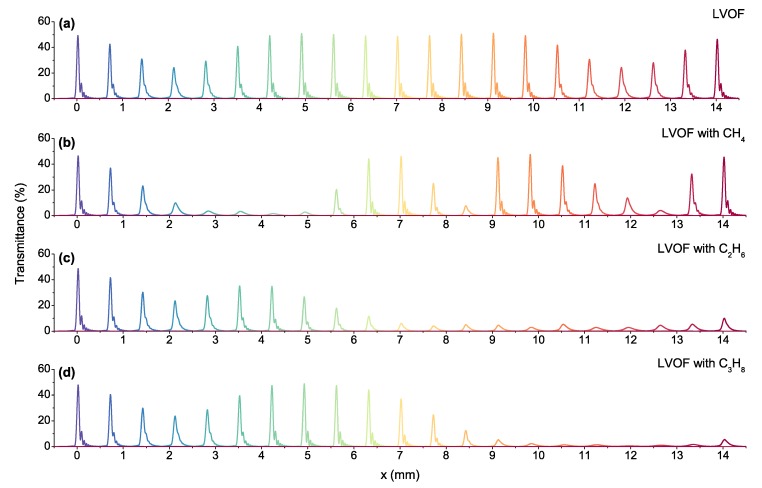
Simulated wideband spectral response of (**a**) the LVOF; (**b**) LVOF with methane; (**c**) LVOF with ethane and (**d**) LVOF with propane.

The spectral response of a 14 mm long gas-filled LVOF that operates in the 3.2 μm to 3.4 μm wavelength range is simulated. The cavity length range of 24 μm to 25.5 μm corresponds to a mirror angle of ϕ=6.1 mdeg, while the distance between the imaginary wedge apex and the onset of the filter is $x_{0}=224\;mm$. The combined reflectivity and transmissivity of the mirrors are calculated using the transfer-matrix method, while taking the thick Si wafers holding the top and the bottom mirrors into account. The thickness of the tapered SiO_2_ layer is centered at 3.3 μm as three quarter-wavelengths to create the 1.5 μm level difference required for the cavity. Therefore, the physical thickness of the SiO_2_ layer with a refractive index of $n_{{SiO}_{2}}=1.44$ varies from 2.46 μm to 0.96 μm. The rest of the mirror layers are quarter-wavelength thick, calculated according to the center wavelength of 3.3 μm. The wideband spectral response of the LVOF is calculated with 1 μm step size in the *x*-axis as shown in Figure 3a. The wavelength is swept from 3.2 μm to 3.4 μm with 10 nm steps, where the response of the filter to the shortest wavelength is located at the onset (x=0 mm). The spectrum is observed right after the bottom mirror, i.e., z=0 mm and the incidence angle is selected as $\theta =-1.61^{\circ }$. The variation between 24.3% and 51% in the peak transmittance is caused by the position-dependent mirror reflectivity. The simulated full width at half maximum (FWHM) resolution is 0.8 nm on average.

High-resolution absorption coefficient spectra of methane, ethane and propane are adapted from the Pacific Northwest National Laboratory (PNNL) vapor phase infrared spectral library [25]. The data is evaluated for 1 atm unit pressure and 1 mm absorption path length in the 3.2 μm to 3.4 μm wavelength range. The resolution of the spectra is 0.112 ${cm}^{-1}$, which translates into 0.122 nm at 3.3 μm center wavelength.

Wideband spectra of LVOF with 100% concentration of methane, ethane and propane are given in Figure 3b–d. High frequency oscillations in the absorption spectrum of methane causes changes in the entire spectrum. Ethane has absorptive features starting at 3.25 μm that confirms the similarity between the spectral response of the empty filter and the LVOF with ethane in the x= 0–3 mm position range. As the wavelength increases, ethane starts to absorb with dominating features between 3.3 μm and 3.4 μm. This wavelength range corresponds approximately to x= 7–14 mm position range, justifying the suppressed transmission curves in Figure 3c. Propane has negligible absorption in the 3.2 μm to 3.3 μm wavelength range. In contrast to methane and ethane, propane has a nonoscillating absorption spectrum, which is less affected by the resolving power of the spectrometer. Similar to ethane, the transmission curves are highly suppressed in the x= 9–14 mm position range; however, the window between x=4 mm and x=9 mm provides distinctive features to distinguish these two gases.

The peak transmittance and resolution of the transmission curves can be improved with oblique incidence due to the nonparallel configuration of the mirrors [26]. At every detector plane position (*z*), there is an incidence angle that provides the identical optimum transmission for a specific wavelength [27]. For the LVOF design in the 3.2 μm to 3.4 μm wavelength range, this angle of incidence varies between −1.38${}^{\circ }$ and −1.85${}^{\circ }$ at z=0 mm. Since a single light source is to be combined with the gas-filled LVOF, the average of these incidence angles, i.e., $\theta =-1.61^{\circ }$ is selected as the optimum value at z=0 mm.

**Figure 4:**
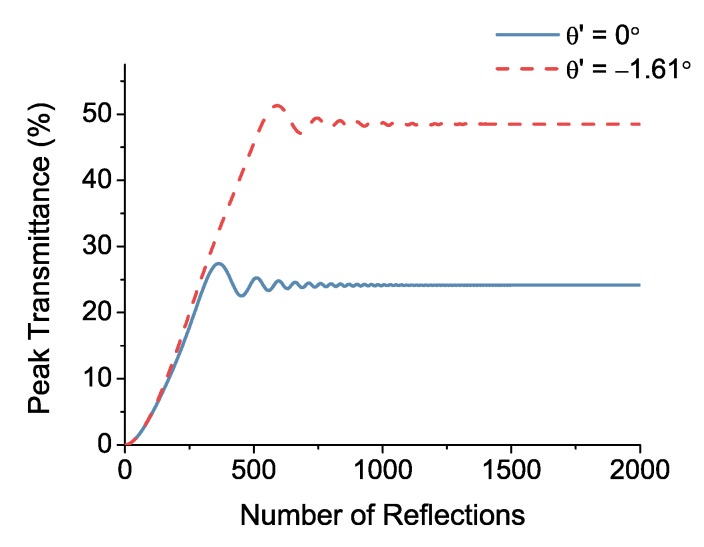
Peak transmittance of the LVOF at 3.3 μm wavelength with respect to the number of reflections in the cavity, observed at normal and oblique incidence.

How does optimizing the angle of incidence affect the performance of the gas-filled LVOF? A filter with better resolution is capable of detecting finer features in the spectrum of the sample, thereby improving the selectivity of the spectrometer. Moreover, a transmission curve with a higher peak value requires a higher number of reflections to reach a steady-state; hence, it elongates the absorption path. To demonstrate the effect of the incidence angle on the number of reflections, the peak of the transmission curve at 3.3 μm is selected. The peak transmittance is estimated at each step by adding the current transmitted wave to the previous set of transmitted waves and calculating the intensity. The number of reflections is related to the number of transmitted waves (*p*) by the expression 2p−2. Figure 4 shows the gradual development of the transmittance with respect to the number of reflections. The comparison between normal incidence and oblique incidence at the optimum value confirms that a higher number of reflections is required to reach a steady-state in the latter. 

As illustrated in Figure 4, after a specific number of reflections, the transmitted waves do not contribute to the interference pattern anymore. Therefore, the optical path difference between the first and the *p*th transmitted wave, i.e., $\Delta S_{p}$, which goes to infinity in theory, cannot be employed as the absorption path length. An effective value for the optical absorption path length ($L_{eff}$) must be calculated using Equation (4), where the transmission response of the empty LVOF is used as a reference to separate the absorption from the wavelength selection:

(4) Leff=lnTLVOFTLVOFTGas−filledLVOFTGas−filledLVOFαc.

**Figure 5:**
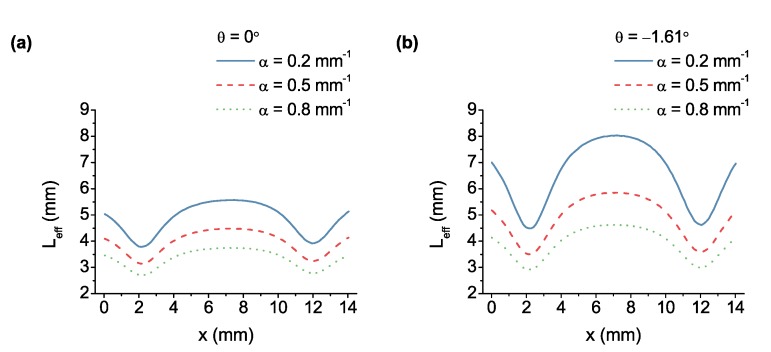
Effective optical absorption path length values calculated along the length of the filter for the absorption coefficients of 0.2 ${mm}^{-1}$, 0.5 ${mm}^{-1}$ and 0.8 ${mm}^{-1}$ at (**a**) normal and (**b**) oblique incidence.

The wideband transmission response of the gas-filled LVOF is calculated for various absorption coefficients at both normal and optimum angle of incidence. The effective absorption path length is extracted using Equation (4) as shown in Figure 5, where full concentration of the sample is assumed and peak transmittance values are compared. The shape of the curve is dominated by the position-dependent reflectivity of the mirrors. Compared to Figure 5a at normal incidence, Figure 5b at the optimum angle of incidence exhibits higher $L_{eff}$ values for each absorption coefficient, affirming the improving effect of the incidence angle on elongation.

The increasing absorption coefficient of the sample at the same concentration results in a shorter effective absorption path at both normal and oblique incidence as shown in Figure 5. Since the optical absorption is incorporated into the operation of the LVOF, the high absorption of the sample dominates the effective number of cavity passes; hence, it shortens the absorption path. As a consequence, the same transmittance can be achieved by different combinations of concentration and absorption coefficient. The wideband operation of the device becomes the key factor in the analysis here. By combining spectral data measured over the entire operating wavelength range and using multivariate analysis methods, the composition of the sample can be extracted despite the absorption coefficient dependency of the effective optical path length.

## 3. Results and Discussion

**Figure 6:**
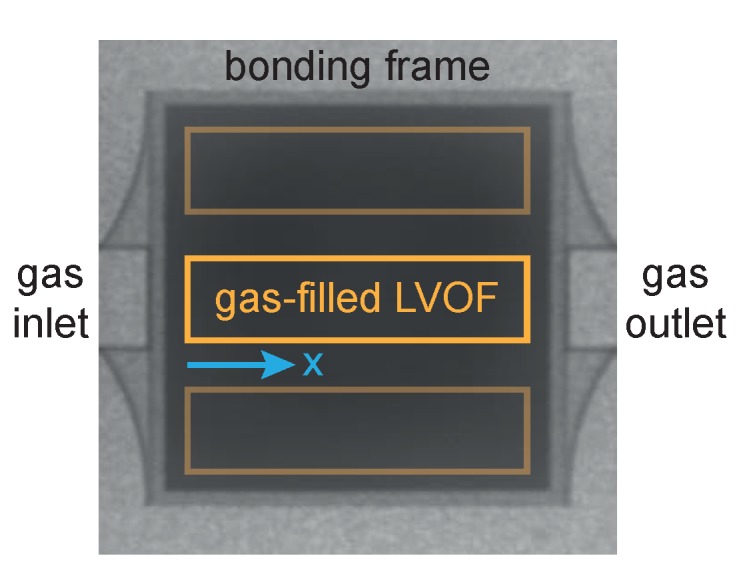
Top-view image of the fabricated device captured by a near-infrared camera.

Gas-filled LVOFs are fabricated in a CMOS-compatible process by combining a flat and a tapered mirror by wafer bonding. The flat mirror is sputtered in a KOH etched cavity to define the cavity length together with the level difference formed by the tapered mirror. The first SiO_2_ layer of the tapered mirror is fabricated by the thermal-chemical reflow of a photoresist composed of variable distanced trenches, followed by one-to-one transfer etching [28]. The level difference of the tapered layer is increased during fabrication to account for the process variations. Thus, the spectral response in the 3.2 μm to 3.4 μm wavelength range is observed along a shorter part of the filter length. Figure 6 shows the top-view image of the fabricated device captured by a near-infrared camera. The 20 mm by 20 mm die consists of three gas-filled LVOFs with gas inlet and outlet on the sides. 

**Figure 7:**
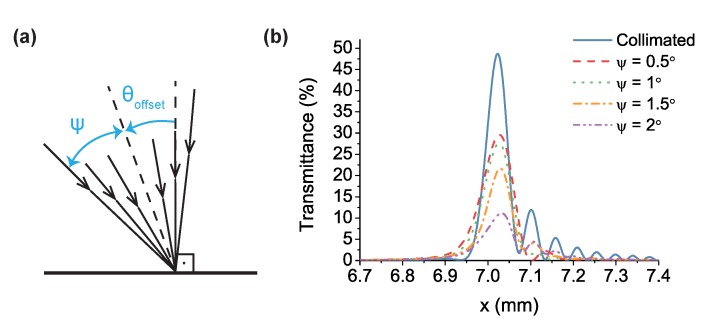
(**a**) the illustration of a light bundle impinging on the flat mirror of the LVOF; (**b**) transmission response of the LVOF calculated for both collimated and non-collimated light at various cone angles.

The nonparallel configuration of the mirrors in the gas-filled LVOF, combined with the high-order operation and highly reflective mirrors, renders the degree of collimation of the light beam a design parameter. The effect of collimation can be simulated by defining the input light as a bundle with a specific half-cone angle, ψ as shown in Figure 7a. By sweeping the incidence angle θ, from −ψ to ψ and calculating the interference of all these transmitted waves, the effect of cone angle on the optical performance is estimated. Moreover, introducing an offset ($\theta_{offset}$) on the incidence angle allows the collection of rays that have an optical performance close to the optimum. 

The spectral response of the filter is simulated at 3.3 μm wavelength for both collimated and non-collimated light at various cone angles. Figure 7b shows the transmission curve with collimated input at the optimum incidence angle of $\theta =-1.61^{\circ }$ in addition to the curves with an offset of $\theta_{offset}=-0.5^{\circ }$ at half-cone angles $\psi =0.5^{\circ }$, $1^{\circ }$, $1.5^{\circ }$ and $2^{\circ }$. Increasing cone angle causes a wider transmission curve with lower maximum transmittance. As demonstrated in Figure 7b, a light source with a half-cone angle of $\psi \leq 1.5^{\circ }$ would provide an acceptable spectral performance. It must be noted that the highest reduction in spectral response is observed at the center wavelength of 3.3 μm, where the mirror reflectivity is the highest.

**Figure 8:**
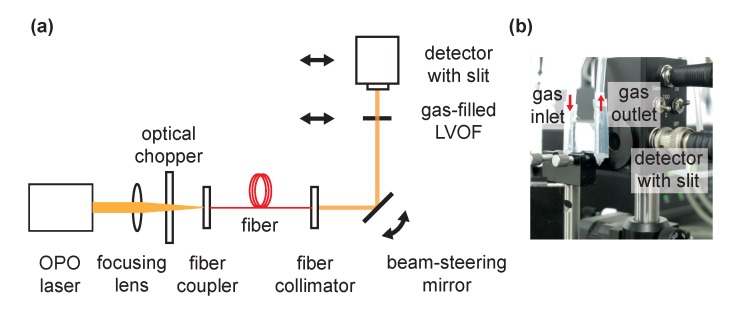
(**a**) schematic illustration of the optical characterization setup; (**b**) filter and detector shown in detail.

The gas-filled LVOF is to be integrated with a miniaturized mid-infrared emitter and a dedicated collimator that together fulfill the requirements of the light source. Since collimation is more effective as the emitter becomes smaller, a mid-infrared light-emitting diode (LED) enables an efficient collimation due to its high-throughput per unit emission area [29,30,31]. Combined with an aspheric [32] or a parabolic collimator [33], an LED-based light source would be compatible with the requirements of the gas-filled LVOF. However, to be able to measure the spectral performance of the LVOF, a measurement tool with better resolution than the filter must be employed. Moreover, the angle of incidence and the position of the detector plane with respect to the filter become crucial parameters due to the nonparallel configuration of the mirrors. Therefore, an optical characterization setup with a high degree of freedom was built using a collimated tunable mid-infrared optical parametric oscillator (OPO) laser as a light source, as shown in Figure 8. 

The output of the experimental OPO laser is focused on the fiber coupler, while passing through an optical chopper operating at 1.6 kHz. After propagating through a single mode ZrF_4_ fiber, the light beam is collimated by an aspheric lens package with a focal length of f=5.95 mm resulting in a light beam with a $1/e^{2}$ diameter of 1 mm at a distance equal to the focal length of the lens. Measured full-angle beam divergence is 0.125${}^{\circ }$, which falls within the $3^{\circ }$ full-cone angle limit of the gas-filled LVOF. The collimated light beam reflects off the beam-steering mirror that is aligned at the optimum incidence angle for the given filter-detector separation. After passing through the device, the light is collected at the large-area PbSe detector, on which a 3 mm-tall and 15 μm-wide slit is mounted to replicate a pixel in a detector array. The combination of a large-area detector and a slit is chosen over a detector array mainly due to the limited availability of an array in the mid-infrared range. Moreover, the constant width of a pixel in such an array could be a limiting factor in characterization, where the detector pixel must be narrower than the length that corresponds to the FWHM resolution of the filter to be able to construct the transmission curve. Using such a slit enables the collection of high-resolution data thanks to its narrow width, while maximizing the light throughput due to its height. The flow of both pure sample gas and nitrogen as the infrared-inactive diluting component through the filter cavity is regulated by mass-flow controllers at a rate of 3 L/h in total.

**Figure 9:**
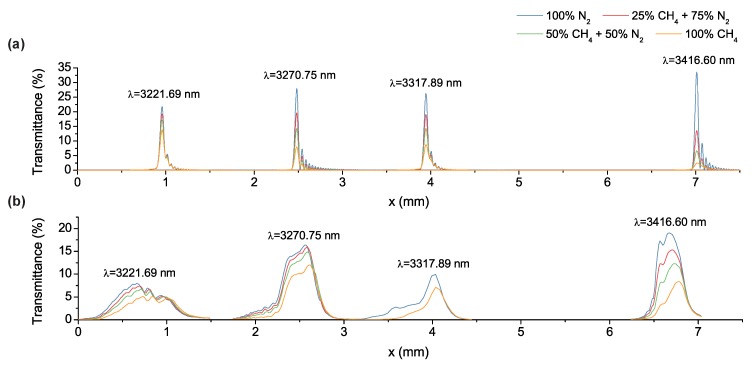
The wideband spectral response of the gas-filled LVOF with various mixtures of methane and nitrogen (**a**) simulated using the measured profile of the mirrors and (**b**) measured using the optical parametric oscillator (OPO) laser at the wavelengths of 3221.69 nm, 3270.75 nm, 3317.89 nm and 3416.60 nm.

The measurement procedure started with tuning the wavelength of the laser by choosing the right slab on the nonlinear crystal and subsequent computer-controlled temperature setting [34]. Then, the position of the transmission curve at this wavelength was approximately located along the length of the device. Later, the incidence angle was adjusted for optimum transmission while 100% nitrogen was flowing through the resonator cavity. To calculate the transmittance, the filter was first taken out of the optical path and a reference measurement was performed. Next, the filter was brought back into the optical path and scanned along its length with 15 μm steps. The ratio of the filter measurement to the reference measurement gave the transmittance. To see the effect of the gas, both pure sample gas and various mixtures diluted with nitrogen were fed into the cavity. Figure 9b shows the wideband spectral response of the gas-filled LVOF measured with methane as the sample gas. 

An experimental optimum incidence angle of $\theta =-6^{\circ }$ at a filter detector distance of z=7 mm was achieved, while the OPO laser was tuned to a wavelength of 3416.60 nm. Despite being out of the operating range, 3416.60 nm was selected due to the high stability of the laser in terms of both wavelength and amplitude over time. The safety margin left for the level difference during fabrication and the repetitive nature of the spectral response in an LVOF nevertheless enabled the capturing of the transmission curve for the 14th operating order. Figure 9b shows the transmission curve at around x=6.6 mm with pure nitrogen and methane as well as their mixtures. The reference transmission curve with nitrogen exhibits a peak transmittance of 18.88%, while the FWHM resolution is 309.68 μm in length. This translates into a 10 nm experimental FWHM resolution in wavelength.

When the gas-filled LVOF is combined with a wideband light source, the resolving power of the sensor is determined by the resolution of the filter. However, in the experimental setup, the laser, which has a 10 MHz linewidth that is equivalent to 363 fm at 3.3 μm wavelength, theoretically outperforms the filter in terms of resolution. The PNNL library contains spectral data for the hydrocarbons investigated in this paper with the highest resolution available in the entire wavelength range of interest. Hence, the absorption coefficient of methane is adapted directly from the PNNL database without any adjustment as $\alpha =0.6120\;{mm}^{-1}$. Using Equation (4) with the peak transmittance ratio of pure nitrogen and methane at 3416.60 nm wavelength, an effective optical path length of 1.33 mm is calculated. This is equivalent to an elongation of 56 times compared to the 23.916 μm-long physical cavity.

The remaining measurements were performed at 3221.69 nm, 3270.75 nm and 3317.89 nm as shown in Figure 9b. The shape of the curves as well as the peak transmittance and FWHM resolution are different at each wavelength. This is mainly due to the position-dependent optimum incidence angle directly resulting from the position dependency of the mirror reflectivity. During the measurements, the optimum angle was adjusted at 3416.60 nm and the alignment was kept the same for the remaining wavelengths. The change in peak transmittance with the sample on the other hand is a function of the absorption of the sample at that wavelength.

**Figure 10:**
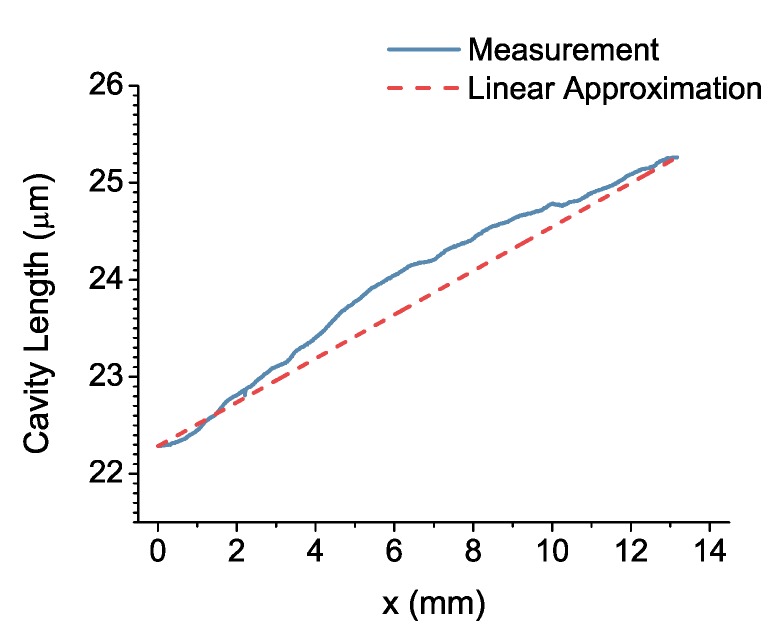
Cavity length calculated using the profile measurements of the flat and the tapered mirrors with the related linear approximation of the slope.

The effect of variations in surface profile of the mirrors; hence, the variations in the cavity length could be incorporated in the Fizeau model either by introducing an error function or by using the measured values in the simulation [35]. Here, the surface profiles of the mirrors are measured using a stylus profilometer and are subsequently included in the mirror reflectivity calculations as well as in the simulation of the spectral response. The wideband spectral response of the filter at an incidence angle of $\theta =-6^{\circ }$ is calculated at z=7 mm as shown in Figure 9a. Measured and simulated spectral responses are in good agreement in terms of the position of the transmission curves. Moreover, the variation in the peak transmittance of the curves in simulations, which is a consequence of the position-dependent reflectivity and the selection of a single optimum incidence angle, matches the characterization results. However, the measured transmission curves are significantly wider than their simulated counterparts, which is due to several practical aspects that have not been taken into consideration. Firstly, the measured profile of the mirrors used in the simulations accounts merely for the length of the cavity and the thickness of the tapered SiO_2_ layer. The change in the angle of reflection as the beam propagates in the cavity is still estimated by a constant slope as shown as the linear approximation curve in Figure 10. Therefore, the localized slope due to the irregularities in the taper is ignored. Moreover, the simulations are based on a single profile measurement taken along the length of the filter. However, the profile variations along the width of the filter contribute to the spectral response as well. Note that the height of the slit placed in front of the detector (3 mm) determines the width of the filter that is covered during the measurements. Furthermore, the angular alignment of the slit on the detector with respect to the filter, combined with the possible variations in profile along the width of the filter could cause widening in the transmission curves.

The effective length of the absorption path is calculated as 4.27 mm using the peak transmittance ratio of pure nitrogen and methane at 3416.60 nm that is simulated with the measured mirror profiles, in Equation (4) with the absorption coefficient adapted from the PNNL library at this wavelength as $\alpha =0.6120\;{mm}^{-1}$. The experimental performance in the elongation of absorption path is therefore reduced. Variations in surface quality of the mirrors and alignment errors in measurement contribute to the elongation performance as well. Moreover, fabrication tolerances could cause changes in the thickness and the refractive index of mirror layers, thereby deviating the actual performance from the design. In addition, the experimental nature of the OPO laser combined with the highly oscillating absorption spectrum of methane might introduce an error in the actual value of the wavelength and the related absorption coefficient.

**Figure 11:**
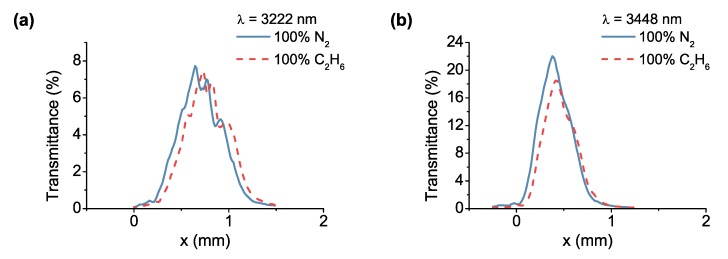
Measured spectral response of the gas-filled LVOF with nitrogen and ethane at the wavelengths of (**a**) 3222 nm and (**b**) 3448 nm.

The spectral response of the gas-filled LVOF to ethane was measured at the wavelengths of 3222 nm and 3448 nm as shown in Figure 11. The alignment of the characterization setup was kept the same as in the methane measurements. Therefore, the incidence angle was selected as $\theta =-6^{\circ }$, while the transmission was observed at z=7 mm. The filter response was captured at the 14th order at 3222 nm wavelength, whereas the 13th order was measured at 3448 nm, confirming the positional distribution of the curves. The change in the transmittance at 3222 nm wavelength is negligible due to the small absorption coefficient ($\alpha =0.0035\;{mm}^{-1}$) of ethane. At 3448 nm wavelength, on the other hand, ethane absorbs relatively more light due to the higher absorption coefficient of $\alpha =0.15\;{mm}^{-1}$. 

In both measurements with methane and ethane, the transmission curves with an absorbing sample in the cavity were shifted along the *x*-axis. This shift could be explained by the theoretical approach based on the Fizeau model, where absorption of the sample is incorporated. Including the effect of absorption in the calculation of the overall transmittance of the gas-filled LVOF using Equation (3) forces a new position for the maximum transmittance due to the *x*-position dependency of the exponential function. Similar shifts were observed in simulations with constant mirror reflectivity along the length of the filter. Additionally, in our approach, where the first SiO_2_ layer is tapered to form a tapered cavity, mirror reflectivity and transmissivity contribute to the positional shift of the curve in the *x*-axis as well, due to their position dependency.

## 4. Conclusions

We have demonstrated a highly miniaturized wideband optical filter with a functionally integrated gas cell. A theoretical framework based on the Fizeau interferometer model, where the effect of the absorbing species is incorporated, was described for spectral analysis. The spectrum of a 15th order gas-filled LVOF was calculated for methane, ethane and propane in the 3.2 μm to 3.4 μm wavelength range using the theoretical description. Spectral distribution of the absorption features of these gases confirmed the selectivity of the gas-filled LVOF. Moreover, we showed that the sensitivity of the device could be improved by applying oblique incidence.

Measurements with methane and ethane in the mid-infrared showed sufficient sensitivity and confirmed the selectivity of the sensor to different hydrocarbons. A quantitative investigation of methane absorption revealed that an effective optical absorption path of 1.33 mm was achieved. This translates into an experimental elongation of 56 times compared to the 23.916 μm-long physical cavity.

The gas-filled LVOF is eligible to be monolithically integrated with a detector array and electronics thanks to its CMOS-compatible bottom-up fabrication approach. Combined with the effective use of the optical resonator as a gas cell, the gas-filled LVOF enables the ultimate miniaturization and functional integration of the gas absorption spectrometer.
